# Brief memory reactivations induce learning in the numeric domain

**DOI:** 10.1038/s41539-022-00136-9

**Published:** 2022-08-17

**Authors:** Gilad Schrift, Dror Dotan, Nitzan Censor

**Affiliations:** 1 School of Psychological Sciences and Sagol School of Neuroscience, Tel Aviv University, Tel Aviv, 69978 Israel; 2 School of Education and Sagol School of Neuroscience, Tel Aviv University, Tel Aviv, 69978 Israel

**Keywords:** Human behaviour, Consolidation

## Abstract

Learning of arithmetic facts such as the multiplication table requires time-consuming, repeated practice. In light of evidence indicating that reactivation of encoded memories can modulate learning and memory processes at the synaptic, system and behavioral levels, we asked whether brief memory reactivations can induce human learning in the numeric domain. Adult participants performed a number-fact retrieval task in which they learned arbitrary numeric facts. Following encoding and a baseline test, 3 passive, brief reactivation sessions of only 40 s each were conducted on separate days. Learning was evaluated in a retest session. Results showed reactivations induced learning, with improved performance at retest relative to baseline test. Furthermore, performance was superior compared to a control group performing test-retest sessions without reactivations, who showed significant memory deterioration. A standard practice group completed active-retrieval sessions on 3 separate days, and showed significant learning gains. Interestingly, while these gains were higher than those of the reactivations group, subjects showing reactivation-induced learning were characterized by superior efficiency relative to standard practice subjects, with higher rate of improvement per practice time. A follow-up long-term retention experiment showed that 30 days following initial practice, weekly brief reactivations reduced forgetting, with participants performing superior to controls undergoing the same initial practice without reactivations. Overall, the results demonstrate that brief passive reactivations induce efficient learning and reduce forgetting within a numerical context. Time-efficient practice in the numeric domain carries implications for enhancement of learning strategies in daily-life settings.

## Introduction

Throughout a wide range of research disciplines extending from cognitive neuroscience to neurorehabilitation and daily-life activities, the prevailing dogma is that learning is enabled by extensive skill execution, known as ‘practice makes perfect’^1,2^. For instance, numerous studies have shown a significant relationship between the time that students spend in learning and their mathematical performance, with performance improving as students’ learning time extends^3,4^. This dogma as a unitary route for learning has been recently challenged by behavioral studies showing that procedural skill learning can be achieved without extensive exercise but rather via brief reactivations of the learned task^5,6^. At the neural level, it has been shown that distinct content-sensitive patterns of neural activity that occur during a perceptual experience, recur when the experience is reminded^7–9^ and predict the consequential memory retrieval^9–16^.

Potentially related to memory-reactivation learning is the phenomenon of memory reconsolidation^17–19^. As stabilized memories are reactivated, either by retrieval or by a reminder cue, they revert to a labile state. Reconsolidation is the process that follows in which these memories are re-stabilized and are susceptible to modifications by incoming inputs. Accumulating evidence indicates that these existing memories can be reinforced, degraded, or updated by the inclusion of new information during the offline reconsolidation phase^5,6,16–29^. With research originating from rodents fear-conditioning frameworks extending to human skill learning, evidence supporting the dynamics of the reconsolidation phase now subsists at the synaptic, systems and behavioral levels.

Here, we set to investigate whether memory-reactivations can induce learning in daily-life settings. To address this question, we embedded the framework of memory reactivations into the field of numerical cognition, specifically that of numeric facts learning.

The process of learning arithmetic facts, such as the multiplication table, consists of at least two aspects. One aspect refers to the mathematical meaning of numeric facts, which involves conceptual learning and may also be tightly related with the development and use of various strategies (such as repeatedly adding a number to calculate a multiplication product, or breaking down the exercise - e.g., 6 × 5 = 5 × 5 + 5). The present study focuses on another aspect – learning the arithmetic facts via rote memorization. This aspect is highly important too, since most educated adults eventually come to learn the multiplication facts by heart, and solve most single-digit multiplication problems not by computing them mathematically or by using rules, but by retrieving a memorized response^30^. Furthermore, multiplication facts are memorized in verbal memory^31–33^, and are in general dependent on language skills and memory^34,35^. Thus, while understanding the mathematical aspects of multiplication has a role in the learning process, memorization is necessary to achieve proficiency and automaticity or fluency, which refers to the ability to recall basic arithmetic facts in a precise way with little effort^36^. Indeed, findings from research of mathematical education show the benefits of achieving fluency in basic mathematical facts^36–40^, and it is perhaps essential in order to develop estimation and mental computation skills^41–45^. However, many students show substantial individual differences and have difficulties in achieving fluency in basic arithmetic facts retrieval, such as multiplication facts^41,42,46–48^. If reactivation-induced learning enables improved fluency in retrieving numeric facts, it could prove as a time efficient type of practice in the numeric domain, carrying potential implications for daily-life practice strategies including in learning disorders caused by neurological conditions.

**Fig. 1:**
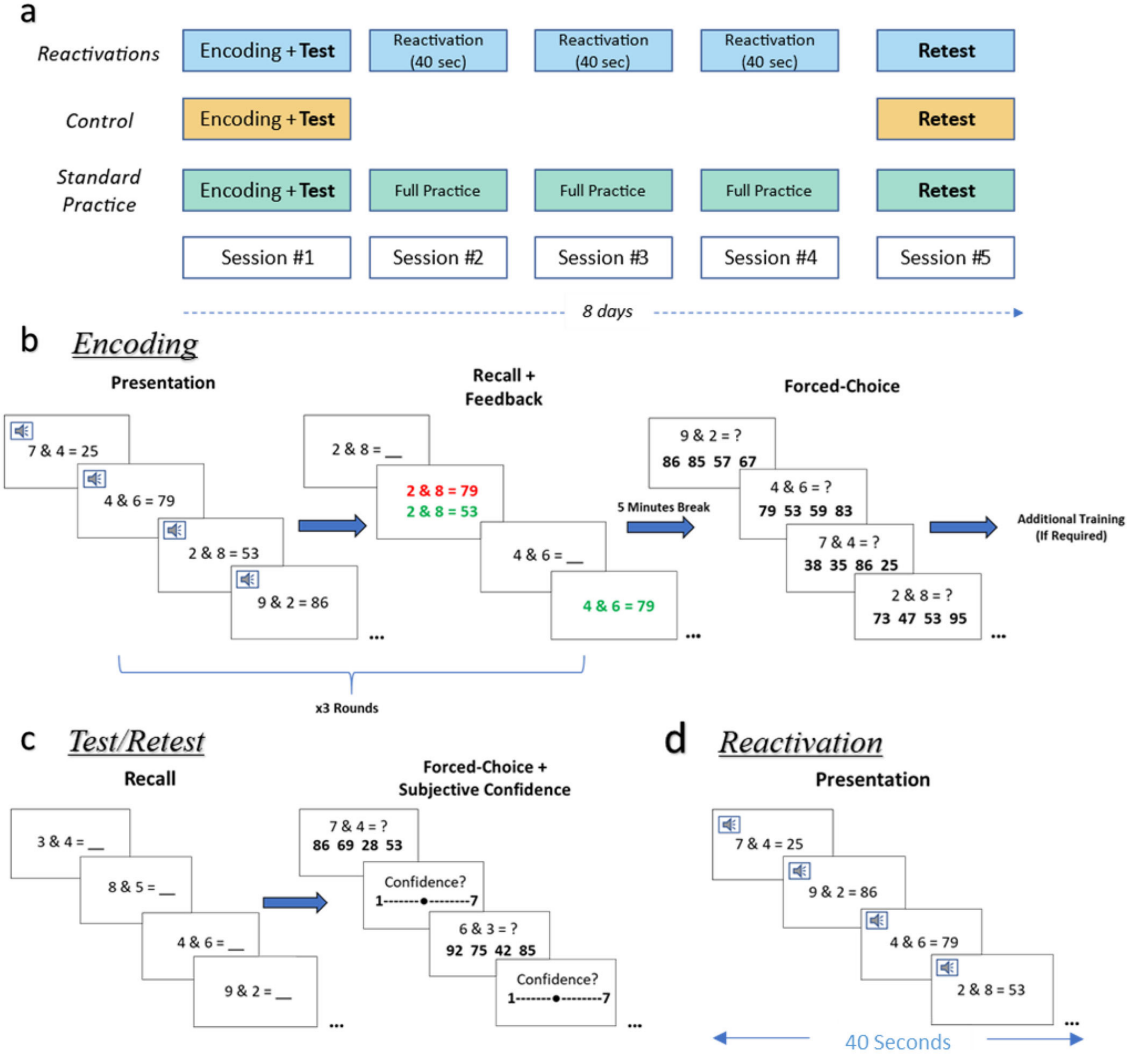
Number-fact retrieval task. **(a)** Experimental design. Participants first encoded the facts and were tested for baseline performance following 30 minutes. Participants in the *Reactivations* group performed 3 brief reactivation sessions in which their memory was briefly reactivated for only 40 seconds of passive facts presentation. The *Control* group performed the test and retest sessions without reactivations. Participants in the *Standard Practice* group performed 3 full practice sessions between test and retest, each containing 3 rounds of facts presentation and recall with feedback. Retest sessions were conducted 8 days following the 1^st^ session. **(b)** Encoding Session: included 3 rounds of active-retrieval practice. Each round consisted of a presentation stage and a recall stage with feedback (correct response = green, wrong response = red accompanied with the correct answer). Participants were asked to fill-in the result as accurately and as fast as they can within a time limit of 8 seconds. Following a 5-minute break, they solved each fact as a 4-alternative forced-choice question in order to determine if an additional training round is required for efficient encoding. Accordingly, an additional practice round was performed for facts with incorrect forced-choice answers. **(c)** Test/Retest Session: consisted of a free recall round, followed by a forced-choice questions round. After each forced-choice question, participants rated their subjective confidence in their answer. **(d)** Reactivation Session: consisted of five seconds of a visual-auditory presentation of each fact in random order, for a total of 40 seconds.

To evaluate the extent of numeric facts learning by memory-reactivations in a population which is already familiar with elementary arithmetic, we used a number-fact retrieval task. In this task, participants had to learn the result of 8 novel numeric facts generated according to specific principles (see Methods). Participants had to later recall these results as quickly and accurately as they can. The numeric facts were first taught and tested in an initial encoding-test session, with a retest session conducted 8 days later (Fig. 1a**)**. Participants in the *Reactivations* group performed brief reactivations over three separate days between encoding-test and retest. Each of these reactivation sessions lasted merely 40 seconds, in which the participants’ memory was passively reactivated by briefly presenting the facts in both auditory and visual modalities. These reactivations were set according to similar studies in the field, and were designed to create a minimal intervention, dissociated from active-retrieval practice, known to affect learning^49,50^, while providing sufficient perceptual information to reactivate and strengthen the existing memory trace^5,6^. The *Control* group performed the encoding-test and retest sessions, without memory reactivations. Participants in the *Standard Practice* group performed three active-retrieval practice sessions between encoding-test and retest, each similar to the day 1 encoding session. Learning gains were quantified as the difference in performance between the baseline test and the retest sessions. In an additional experiment, we examined whether such a form of rapid learning with brief exposures can enable efficient long-term retention following initial standard problem-solving practice.

## Results

### Experiment 1 – reactivation-induced learning

**Fig. 2:**
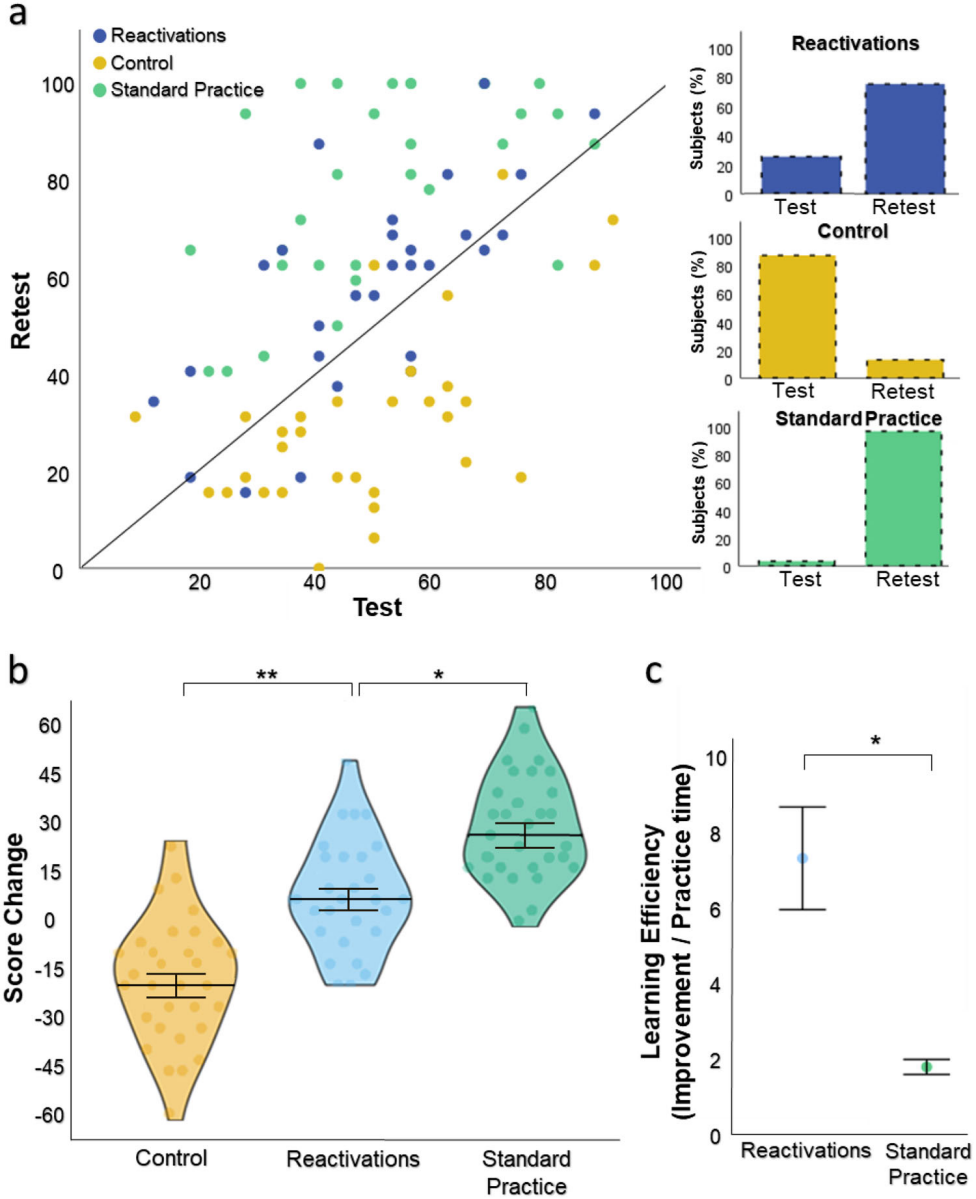
Reactivation-induced learning gains. **(a)** Baseline test versus retest single-subject performance score, presented in a scatterplot along a unit slope line (y = x), where each point reflects a participant. Data accumulating above the unit line reflect participants who improved from test to retest, expressing learning gains, while data points below the line indicate degraded retest performance. Colored bars reflect the percentage of participants on each side of the unit slope line. **(b)** Violin graph showing the mean score change from test to retest sessions. Each point reflects a participant. **(c)** Subjects showing reactivation-induced learning were characterized by superior learning efficiency relative to *Standard Practice* subjects showing learning, with higher rate of improvement (percent) per practice time (minutes). * *P* < 0.0002, ** *P* < 10^−6^. Error bars represent standard error of the mean.

To verify that there were no baseline differences between groups, we conducted a one-way ANOVA of the baseline test sessions. No group effect was found on the participants’ recall score (F_2,86_ = 0.135, *P* = 0.87), reaction times (RTs) (F_2,86_ = 1.083, *P* = 0.34), forced-choice correct answers (F_2,86_ = 0.219, *P* = 0.80) or level of confidence (F_2,86_ = 1.196, *P* = 0.31). We then continued to test whether the brief reactivations induced learning gains. A repeated measures ANOVA on the participants’ score, with Session (baseline, test) and Group (*Reactivations, Control, Standard Practice*) as factors, showed significant Session (F_1,86_ = 10.518, *P* = 0.002) and Session x Group interaction effects (F_2,86_ = 52.413, *P* < 10^−14^). Post-hoc paired-samples t-tests verified that the *Reactivations* group obtained significant learning gains (mean test-retest score change = 8.48 ± 2.94 S.E., t(27) = 2.89, *P* = 0.008), in contrast to the *Control* group (Post-hoc Tukey test, *P* < 10^−6^, Bonferroni corrected) whose performance deteriorated (mean score change = −18.04 ± 3.15 S.E., t(30) = −5.72, *P* < 10^−5^) (Fig. 2a, b). The *Standard Practice* group showed the largest learning gains (mean score change = 27.71 ± 3.39 S.E., t(29) = 8.18, P < 10^−8^). Learning gains of the Standard Practice group were considerably higher than those of the Reactivations group (Post-hoc Tukey test, *P* = 0.0001, Bonferroni corrected). As per the primary hypothesis, a key advantage of brief reactivations is their ability to induce learning with minimal practice time, in contrast to full standard practice^5,6^. Consistently, we measured practice efficiency, defined as the amount of improvement achieved between baseline to retest divided by the practice time spent in minutes (see Methods). A one-way ANOVA between groups examining the practice efficiency revealed that subjects who exhibited reactivation-induced learning (n = 21) were characterized by superior efficiency relative to the standard group subjects who showed learning (n = 29; F_1,48_ = 22.73, *P* = 0.00002) (Fig. 2c).

A repeated measures ANOVA on the participants’ RTs showed a significant Session (F_1,86_ = 14.613, *P* = 0.0003) and Session x Group interaction effects (F_2,86_ = 7.688, *P* = 0.0009). Paired-samples t-tests tests revealed that the *Reactivations* group had a significant reduction in reaction times (mean test-retest RT change = −487ms ± 174 ms S.E., t(27) = −2.80, *P* = 0.009), while the *Control* group did not (mean change = 0.124 ms ±177 ms S.E, t(30) = 0.70, *P* = 0.49). RT reduction was also observed for the *Standard Practice* group (mean change = −867ms ± 194 ms S.E, t(29) = −4.47, *P* = 0.0001).

A repeated measures ANOVA on FC performance also showed a significant Session x Group interaction (FC correct answers: F_2,86_ = 46.483, *P* < 10^−13^; FC confidence: F_2,86_ = 26.496, *P* < 10^−8^). Paired-samples t-tests revealed that the *Reactivations* group maintained their number of FC correct answers and confidence (mean test-retest FC correct answers change = 0.429 ± 0.24 S.E., t(27) = 1.76, *P* = 0.09; confidence change = 0.31 ± 0.18 S.E., t(27) = 1.71, *P* = 0.1) while the *Control* group showed significant deterioration (correct answers change = −1.96 ± 0.27 S.E., t(30) = −7.33, *P* < 10^−7^; confidence change = −1.02 ± 0.18 S.E, t(30) = −5.74, *P* < 10^−5^). The *Standard Practice* group showed improvement in FC performance and confidence (FC change = 1.47 ± 0.25 S.E, t(29) = 5.81, *P* < 10^−5^; confidence change = 1.01 ± 0.24 S.E, t(29) = 4.24, *P* = 0.0002).

In sum, the results indicate that brief reactivations induced significant learning gains and prevented memory deterioration, which was significant in the absence of reactivations. In addition, while learning gains were higher following standard practice, subjects who benefited from reactivation-induced learning were characterized by superior efficiency relative to standard practice subjects, with higher rate of improvement per practice time.

### Experiment 2 – long-term retention

**Fig. 3:**
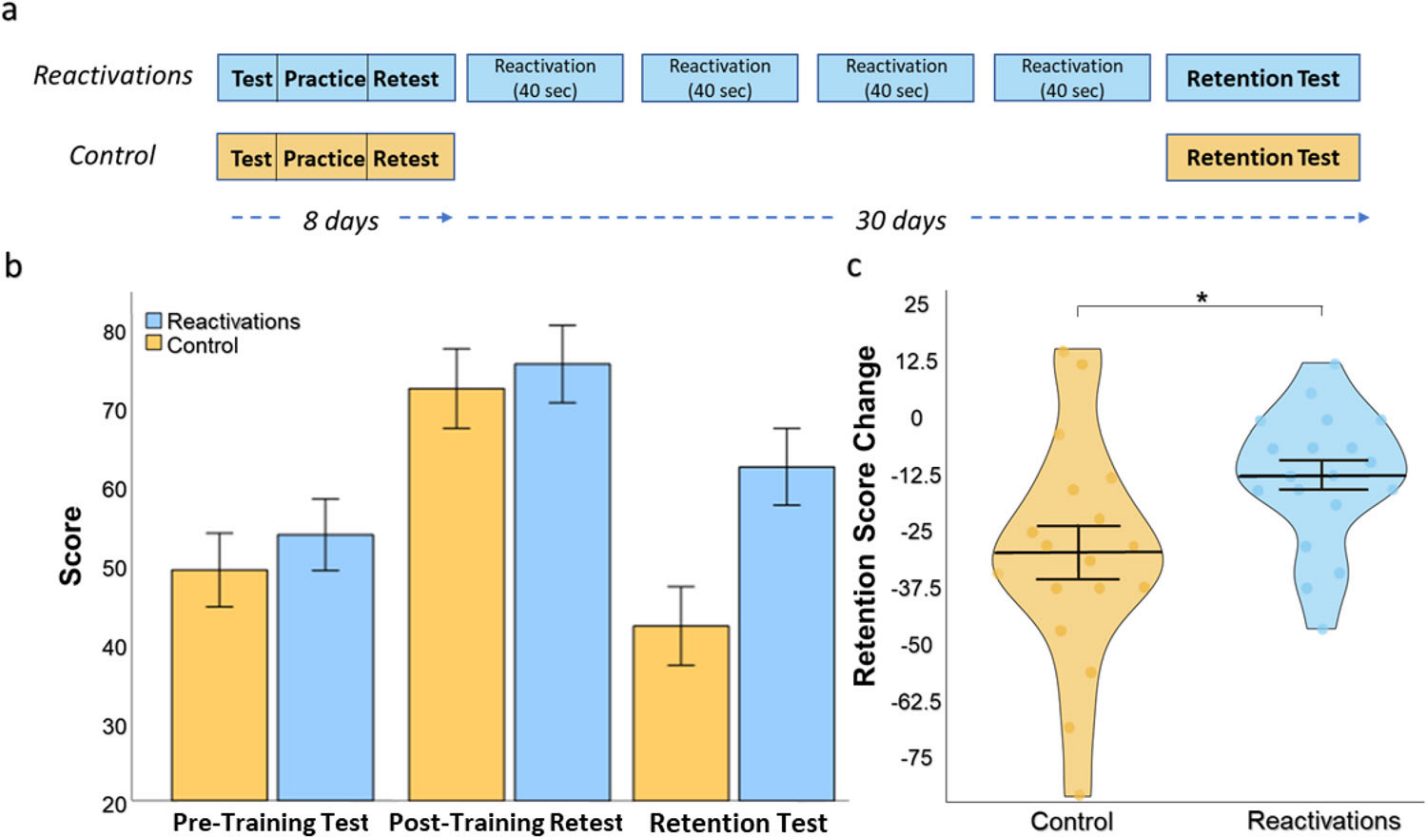
Long-term retention. **a** Experimental design. Participants first completed an 8-day standard practice procedure and were tested for retention following 30 days. Participants were divided into 2 groups: *Reactivations* which completed 40-second reactivation sessions once per week between post-training retest and retention test, and *Controls* without reactivations. **b** Mean scores per session for each group. **c** Violin graph showing the mean score change from post-training retest to retention. Each point reflects a participant. *P < 0.02. Error bars represent standard error of the mean.

To examine the scope of the reactivations effect, in particular whether reactivations would be beneficial also for long-term retention (typical for real-life situations of memorizing arithmetic facts), we conducted an additional experiment which tested the ability of reactivations to retain learning over a longer period of time in the absence of additional practice. To address this question, participants first fully learned the task and achieved a high level of performance, via an 8-day (5 sessions) standard practice procedure ending in a post-training retest. Then, a retention test was performed 30 days following the post-training retest. There were 2 groups – *Reactivations* and *Control*. The *Reactivations* group underwent a 40-second reactivation once per week between post-training retest and the retention test. The *Control* group received no training or reactivations during the retention interval (Fig. 3a). Of note, as opposed to experiment 1 which evaluated the impact of standard practice on learning, experiment 2 focused on long-term retention in the absence of additional practice. Accordingly, retention was tested with or without reactivations, and did not include a standard practice group. As expected, a repeated measures ANOVA on the initial training course with Session (*Pre-training test, Post-training retest*) and Group (*Reactivations, Control*) as factors showed no group effect on recall score (F_1,35_ = 0.705, *P* = 0.41), RTs (F_1,35_ = 1.059, *P* = 0.31), FC performance (F_1,35_ = 0.271, *P* = 0.61), and FC confidence (F_1,35_ = 0.033, *P* = 0.86). We then continued to test whether the brief reactivations promoted long-term memory retention. A repeated measures ANOVA on participants’ score with Session (*Post-training retest, Retention*) and Group (*Reactivations*, *Control*) as factors revealed a significant effect of Session (F_1,35_ = 39.91, *P* < 10^−6^). Importantly, a significant Session x Group interaction (F_1,35_ = 6.26, *P* = 0.017) indicated that the decline in performance in the *Reactivations* group (mean post-training retest – retention score change = −12.99 ± 3.48 S.E., t(18) = −3.72, *P* = 0.002, paired t-test) was smaller than in the *Control* group (mean score change = −30.03 ± 5.95 S.E., t(17)= −5.05, *P* = 0.0001, paired t-test), suggesting that weekly reactivations reduced forgetting (Fig. 3b, c).

No significant Session x Group interactions were observed on RTs, FC (F_1,35_ = 0.286, *P* = 0.60; F_1,35_ = 0.722, *P* = 0.40), and a significant interaction was observed on FC confidence (F_1,35_ = 4.213, *P* = 0.048).

## Discussion

In this study, we aimed to reveal whether reactivating a consolidated memory of numeric facts can strengthen it and promote learning gains. The results showed that 40-second visual-auditory reactivations improved recall of the numeric facts, measured 8 days following encoding. Reactivation-induced learning resulted in a significant improvement compared to the baseline test, and was superior to control without reactivations which showed deteriorated performance relative to baseline. Furthermore, while average learning gains induced by reactivations were lower than the standard practice group, subjects showing reactivation-induced learning were characterized by superior efficiency relative to standard practice subjects, with higher rate of improvement per practice time. This efficiency of learning in the reactivations group relative to the standard practice group may be of crucial importance for real-life pedagogical settings. We then initiated an additional experiment, examining whether reactivations can prove beneficial for long-term retention, which is most relevant for real-life learning. First, participants fully learned the task with standard training, and achieved a high level of performance, replicating the standard practice results of Experiment 1. Then, in the subsequent 4 weeks, brief memory reactivations were performed only once per week, followed by a final long-term retention test. The results showed that brief memory reactivations can reduce long-term forgetting of numeric facts compared to controls without reactivations, demonstrating that reactivations can be beneficial not only during learning (Experiment 1), but also for long-term retention (Experiment 2). Thus, reactivation may play a role in both learning and long-term retention.

These findings are in good agreement with an increasing number of studies reporting memory strengthening via reactivations in procedural learning^5,6^. Taken together with experiments showing memory interference effects induced by memory reactivation^51–55^, our findings support the assumption that the reactivation process facilitates learning associated with declarative human memory, and exists also in a mathematical context. The neural mechanisms underlying this novel form of reactivation-induced learning remain to be determined. Recent research points to the existence of an overlapping fronto-parietal network of numerical cognition composed of intraparietal and prefrontal areas^56–58^ This network includes nodes related to the three primary representational codes which exist for numbers according to the prominent Triple Code Model: the visual Arabic number form (e.g., “2”), the auditory verbal word (e.g., “two”), and analogue non-symbolic size representations (e.g., “■■”)^31^. Taken together with evidence showing that following encoding, memories with more neural reactivations during awake rest periods are better remembered^17^, it is conceivable that neural reactivations within this network play a role in the consolidation of hippocampus-dependent memories^59^, and subsequently facilitate reactivation-induced learning of numerical facts.

Throughout this study, the task introduced the participants with arbitrary numeric facts. While participants were told the facts presented will be fictional with no relation to mathematical conventions, it is likely that additional executive functions were required throughout the learning process to inhibit prior knowledge regarding the numbers (for example, inhibiting ‘24’ when learning ‘3 & 8 = 29’). The numeric facts did not involve a systematic rule, enabling a focus on numeric fact memorization during the recall process. However, the cognitive processes accompanying arithmetic learning contain further aspects and are more complex. Therefore, it remains of interest to further understand the interaction between systematic rules involved in arithmetic and reactivation-induced learning.

While the rationale for designing the current study has been primarily based on the reactivation-reconsolidation framework, additional learning mechanisms should be discussed. In this study, memory reactivations were purposefully designed to be performed in a distributed manner, in order to enable postreactivation offline strengthening of the memory trace^5,6^. As distributed training has been shown to affect the learning process^60,61^, this could explain to some extent the learning gains obtained by the reactivations group. Additionally, the full practice group may have benefited not only from practice per se but also from the recurrent test itself^49,50^. Consistent with this view, the reactivation group did not undergo active-retrieval testing, rather only a very brief reminder, resulting in an intermediate level of performance. Future studies should test whether further incorporating spacing and testing effects into the reactivations, or adding more reactivation-reconsolidation cycles, would result in even more efficient learning processes.

In sum, our findings in two separate experiments demonstrate that memory-reactivations, induced by brief audio-visual presentations, can lead to efficient learning as well as to reduced forgetting of numerical facts. This unique form of skill learning approach may also help promote strategies for developing innovative, productive learning practices for numerical fluency. Future research examining the efficacy of reactivation induced learning, for example by applying brief passive exposures to information that was learned in the classroom, may assert its use as a time-efficient tool for skill acquisition, expanding its relevance for daily-life educational settings. Additional research may also reveal whether this form of learning can facilitate implementation of novel learning strategies geared to reduce the requirement for prolonged task execution, potentially beneficial for disorders caused by neurological conditions such as ADHD.

## Methods

### Subjects

The initial experiment (Experiment 1) consisted of 96 naive healthy participants, ages 20-31 years (84 female, mean age 23.5 ± 2.1 s.d.). The follow-up experiment (Experiment 2) consisted of 39 naive healthy participants, ages 20-40 years (29 female, mean age 25.2 ± 2.8 s.d.). All participants gave their written informed consent to participate in the project, which was approved by Tel Aviv University’s Ethics committee. All procedures were in accordance with approved guidelines. To determine the estimated required sample size for the initial experiment, we conducted an a priori power analysis for between factors repeated measures ANOVA for 3 groups with an alpha = .05, power = .95 and an effect size based on pilot studies conducted in the lab (with Cohen’s d = .36), using G*Power3^62,63^. A similar analysis was conducted for the follow-up retention experiment, using an a priori power analysis for between factors repeated measures ANOVA for 2 groups with an alpha = .05, power = .95 and an effect size based on the initial experiment (Cohen’s d = .55). Participants were randomly allocated to experimental conditions, which were conducted in a single-blinded fashion. Participants were required to sleep at least 6 hours the night before each experimental session, which was performed during daytime. All sessions were conducted online using Pavlovia (https://pavlovia.org/). Seven participants from the first experiment (2 *Reactivations*, 4 *Control*, 1 *Standard Practice*) and two participants from the retention experiment (2 *Control)* whose test-retest learning was 1.5 times the interquartile range above the third quartile or below the first quartile, were identified as outliers and excluded from further analysis^64^.

### Number-fact retrieval task

To mimic numeric fact learning within a population of adult participants, who are already familiar with elementary arithmetic, we generated 8 novel numeric facts. This number of facts was determined based on pilot experiments, in order to achieve an average score of ~40% after the first day session, leaving room for further learning in subsequent sessions. In all facts, the operands were single digits and the result was a 2-digit number (which could be prime), with no repeating digit within a fact. Only the digits 2–9 were used for facts generation, with the digits 0 and 1 excluded due to their unique mathematical meaning in arithmetic operations. When generating the facts, we aimed for no obvious relation between the operands and the results of a fact, and for low between-fact similarity that may disrupt learning^65,66^. Participants were told that there was no systematic rule for the facts, which were presented using the nonexistent operator ‘&’ (e.g., “3 & 8 = 29”). All participants learned the same 8 facts. Performance was quantified by the recall score: a correct result digit in the correct position of the result scored 1 point, and each correct digit in the incorrect position scored 0.5 points. Thus, the score for each fact was between 0 and 2. To calculate recall score in a scale of 0-100, the total points were then divided by 16, the maximum possible number of points. Recall response times were also measured. The task was created using PsychoJs^67^.

### Experimental procedure

#### Experiment 1 – reactivation-induced learning

##### Day 1 session, encoding

Memory was initially encoded and consolidated in the Day 1 session, in which the facts were first presented and taught. The session was identical for participants in all groups. Participants were first given instructions on how the task interface works and completed a warm-up exercise (0 & 0 = 0).

They then performed 3 rounds of active-retrieval practice. Each round consisted of a presentation stage followed by a recall stage. In the presentation stage, each item was presented on the screen for 6 s and was simultaneously narrated aloud. In the recall stage, the exercises without the results were presented (visually and auditory) one by one, and the participants were asked to fill-in the result as accurately and quickly as they could within a time limit of 8 s. The correct result was then shown, i.e., feedback was provided for both incorrect and correct responses. The order of facts was random both in presentation and in recall.

After a 5-minute break, participants completed a forced-choice (FC) test in which they were required to choose, for each of the 8 facts, the correct result out of 4 options with no feedback provided. Options consisted of the correct result, a distractor containing one digit from the correct result in the correct position and one irrelevant digit, a distractor containing one digit from the correct result in the incorrect position and one irrelevant digit, and a distractor, which was the result of a different fact. The forced-choice test was included in order to determine if additional training was required for better encoding, and repeated up to 2 times. An additional practice round was given for facts unsuccessfully recalled in the forced-choice test (Fig. 1b).

##### Day 1 session, test

Following a 30-minute break, a free recall baseline measurement was collected, in which the facts were shown in random order, without feedback. In addition, a forced-choice test was administered as described above; the distractors were selected using the same method, but the specific distractors were different from the ones used earlier in the session. After each forced-choice question, participants also rated their subjective confidence in their answer being correct on a visual analog scale of one to seven (Fig. 1c).

##### Day 2-4 sessions, practice

The *Reactivations* group (*n* = 28) sessions included a passive, five second visual-auditory single presentation of each fact in random order, thus each reactivation session was only 40 s in total (Fig. 1d). The *Control* group (*n* = 31) performed only test and retest sessions, without reactivations. The *Standard Practice* group’s (*n* = 30) procedure was identical to the first part of the Day 1 active-retrieval session: it consisted of the 3 practice rounds, but did not include the forced-choice test, the additional practice rounds and the post-practice retest.

##### Day 5 session, retest

Participants returned for a retest session, which was the same as the baseline-test in the 1^st^ day, with different distractors in the forced-choice questions (Fig. 1c**)**.

#### Experiment 2 – long-term retention

In a separate experiment, participants first completed an 8-day standard practice procedure and were tested for retention following 30 days (Fig. 3a). Participants were divided into 2 groups: A *Reactivations* group (*n* = 19) who completed 40-second reactivations once per week between the post-training retest and the long-term retention test, and a *Control* group (*n* = 18) without reactivations. Weekly reactivations were identical to the reactivations in the first experiment. The retention test was similar to the pre-training test and post-training retest, with different distractors for the forced-choice questions.

### Data analysis

To verify that there were no baseline differences between groups in experiment 1, we conducted a one-way ANOVA of the baseline test session for score, RT, and FC performance. Learning gains were quantified as the difference between the test score on session 1 (encoding) and the test score on the last session (retest), and tested via a 2×3 repeated measures ANOVA with time as within-subject factor, and group as between-subjects factor. A similar analysis was performed to account for the difference in RT, and FC performance.

Practice efficiency was defined as the percent of improvement achieved between baseline and retest, divided by the practice time spent in minutes (e.g., a score of “7” reflects an average test-retest improvement of 7% per each minute of practice), and was tested via a one-way ANOVA between subjects exhibiting reactivation-induced learning (*Reactivations)*, and *Standard Practice* learners.

To verify that there were no baseline differences for the standard practice procedure (sessions 1-5) between groups in experiment 2, we conducted a 2×2 repeated measures ANOVA with time as within-subject factor, and group as between-subjects factor for the test score, RT, and FC performance. Long-term retention was quantified as the difference between the test score on session 5 (post-training retest) and the test score following 30 days (retention), and was tested via a 2×2 repeated measures ANOVA with time as within-subject factor, and group as between-subjects factor. A similar analysis was performed to account for the difference in RT and FC performance.

### Reporting Summary

Further information on research design is available in the Nature Research Reporting Summary linked to this article.

## Supplementary information

Reporting Summary Checklist

## Data Availability

The datasets collected and analyzed during the current study are available upon request from the corresponding author.
