# Supplementary material for: Brief memory reactivations induce learning in the numeric domain
Source: NPJ Sci Learn. 2022 Aug 17;7:18. doi: 10.1038/s41539-022-00136-9 (PMC9385657; doi:10.1038/s41539-022-00136-9)
Supplement: Supplementary file 1 — Reporting Summary Checklist [file 41539_2022_136_MOESM1_ESM.pdf]

nature portfolio

Corresponding author(s): Nitzan Censor

Last updated by author(s): May 31, 2022

Reporting Summary

Nature Portfolio wishes to improve the reproducibility of the work that we publish. This form provides structure for consistency and transparency in reporting. For further information on Nature Portfolio policies, see our Editorial Policies and the Editorial Policy Checklist.

Statistics

For all statistical analyses, confirm that the following items are present in the figure legend, table legend, main text, or Methods section.

n/a	Confirmed	
☐	☒	The exact sample size (n) for each experimental group/condition, given as a discrete number and unit of measurement
☐	☒	A statement on whether measurements were taken from distinct samples or whether the same sample was measured repeatedly
☐	☒	The statistical test(s) used AND whether they are one- or two-sided<br>*Only common tests should be described solely by name; describe more complex techniques in the Methods section.*
☒	☐	A description of all covariates tested
☐	☒	A description of any assumptions or corrections, such as tests of normality and adjustment for multiple comparisons
☐	☒	A full description of the statistical parameters including central tendency (e.g. means) or other basic estimates (e.g. regression coefficient) AND variation (e.g. standard deviation) or associated estimates of uncertainty (e.g. confidence intervals)
☐	☒	For null hypothesis testing, the test statistic (e.g. F, t, r) with confidence intervals, effect sizes, degrees of freedom and P value noted<br>*Give P values as exact values whenever suitable.*
☒	☐	For Bayesian analysis, information on the choice of priors and Markov chain Monte Carlo settings
☒	☐	For hierarchical and complex designs, identification of the appropriate level for tests and full reporting of outcomes
☐	☒	Estimates of effect sizes (e.g. Cohen's d, Pearson's r), indicating how they were calculated

Our web collection on statistics for biologists contains articles on many of the points above.

Software and code

Policy information about availability of computer code

Data collection	PsychoJs(https://github.com/psychopy/psychojs); Pavlovia (https://pavlovia.org/)
Data analysis	SPSS statistics 27; Jupyter Notebook; PlotsOfData

For manuscripts utilizing custom algorithms or software that are central to the research but not yet described in published literature, software must be made available to editors and reviewers. We strongly encourage code deposition in a community repository (e.g. GitHub). See the Nature Portfolio guidelines for submitting code & software for further information.

Data

Policy information about availability of data

All manuscripts must include a data availability statement. This statement should provide the following information, where applicable:

- Accession codes, unique identifiers, or web links for publicly available datasets
- A description of any restrictions on data availability
- For clinical datasets or third party data, please ensure that the statement adheres to our policy

The datasets collected and analyzed during the current study are available upon request from the corresponding author.

Field-specific reporting

Please select the one below that is the best fit for your research. If you are not sure, read the appropriate sections before making your selection.

☐ Life sciences ☒ Behavioural & social sciences ☐ Ecological, evolutionary & environmental sciences

For a reference copy of the document with all sections, see nature.com/documents/nr-reporting-summary-flat.pdf

Behavioural & social sciences study design

All studies must disclose on these points even when the disclosure is negative.

Study description	The study is a quantitative experimental study conducted online. Data is quantitative and analyzed accordingly.
Research sample	The sample was assembled from Tel-Aviv university undergraduates and Israeli Facebook users who replied to an open invitation to partake in the experiment. Overall, 135 naive healthy participants aged 18-40 years (113 female; mean age 24.0 ± 2.2 s.d.) were included in the study. The rationale for recruitment was to assure results could be generalized.
Sampling strategy	Sampling procedure was random. Sample sizes were estimated based on power analysis of pilot studies conducted in the lab, using G*Power3.
Data collection	Stimuli were programmed, presented and responses recorded using the Pavlovia platform.
Timing	Experiments were performed during daytime. For the first experiment, data were collected between November 16, 2020 and January 14, 2021; For the follow-up retention experiment, data were collected between June 7, 2021 and September 5, 2021.
Data exclusions	Seven participants from the first experiment and two participants from the follow-up retention experiment whose test-retest learning was 1.5 times the interquartile range above the third quartile or below the first quartile, were identified as outliers and excluded from further analysis. The exclusion criterion was pre-established to adjust for any conditions that may apply to online study participants, resulting in unusual performance.
Non-participation	See above.
Randomization	Participants were randomly allocated to experimental conditions. The number of participants allocated each time altered between 1-4 after each batch of allocations.

Reporting for specific materials, systems and methods

We require information from authors about some types of materials, experimental systems and methods used in many studies. Here, indicate whether each material, system or method listed is relevant to your study. If you are not sure if a list item applies to your research, read the appropriate section before selecting a response.

Materials & experimental systems

n/a	Involved in the study	
☒	☐	Antibodies
☒	☐	Eukaryotic cell lines
☒	☐	Palaeontology and archaeology
☒	☐	Animals and other organisms
☐	☒	Human research participants
☒	☐	Clinical data
☒	☐	Dual use research of concern

Methods

n/a	Involved in the study	
☒	☐	ChIP-seq
☒	☐	Flow cytometry
☒	☐	MRI-based neuroimaging

Human research participants

Policy information about studies involving human research participants

Population characteristics	See Above
Recruitment	See Above
Ethics oversight	Tel Aviv University's Ethics committee

Note that full information on the approval of the study protocol must also be provided in the manuscript.
